# Kidney Function, Age, and Education as Contributors to Depression and Anxiety in Juvenile Systemic Lupus Erythematosus

**DOI:** 10.3390/ejihpe11040107

**Published:** 2021-11-25

**Authors:** Margarida Figueiredo-Braga, Beatriz Silva, Sara Ganhão, Francisca Aguiar, Caleb Cornaby, Iva Brito, Brian D. Poole

**Affiliations:** 1 Faculty of Medicine, University of Porto, 4200-319 Porto, Portugal; ivaobrito@hotmail.com; 2 i3S.Instituto de Investigação e Inovação em Saúde, University of Porto, 4200-319 Porto, Portugal; 3 Young Adult and Pediatric Rheumatology Unit, Centro Hospitalar e Universitário do Hospital de São João, 4200-319 Porto, Portugal; bpfs96@gmail.com (B.S.); sganhaods@gmail.com (S.G.); francisca.ra@hotmail.com (F.A.); 4 Department of Pathology and Laboratory Medicine, University of North Carolina at Chapel Hill, Chapel Hill, NC 27599, USA; caleb.cornaby@unchealth.unc.edu; 5 Department of Microbiology and Molecular Biology, Brigham Young University, Provo, UT 84602, USA; brian_poole@byu.edu

**Keywords:** juvenile systemic lupus erythematosus, lupus, nephritis, depression, anxiety, autoimmune

## Abstract

Juvenile systemic lupus erythematosus (JSLE) is diagnosed in children younger than 18 years of age. Depression and anxiety are common, but not well understood in JSLE. We investigated the clinical and psychological factors associated with the psychological manifestations of JSLE. Twenty-nine JSLE patients were recruited for the study. Patients completed surveys evaluating their psychological status and perceptions about their health. Medical records were used to obtain laboratory results. The JSLE patient population was compared with adult-onset SLE (ASLE) patients and unaffected controls. Kidney involvement was associated with depression in the JSLE patients. The BUN levels, BUN/creatinine ratio, and leukocyturia were all significantly associated with depressive symptoms. Multivariate analysis found that the BUN/creatinine ratio was the most predictive value for both depression and anxiety. Depressive symptoms in JSLE were less pronounced than in ASLE, although anxiety was not different. Age and education are likely to be protective against depression in the JSLE patients. These findings may indicate that symptomatology is an important indicator of whether the patient needs psychiatric care.

## 1. Introduction

Although systemic lupus erythematosus (SLE) is usually thought of as a disease affecting post-adolescent women, up to 20% of individuals with SLE develop the disease before their 18th birthday [1]. This is known as juvenile systemic lupus erythematosus (JSLE). JSLE has several features that distinguish it from adult-onset SLE, including often greater disease severity and a lower female predominance, especially in children less than 5 years old [2]. Organ involvement, including kidney disease, is more common in JSLE than in SLE [3,4].

One of the hallmarks of SLE is neurological and psychiatric symptomatology, especially anxiety and depression. These are not well understood in JSLE patients, although up to 47% of JSLE patients will experience depressive symptoms at some time during their lives [5]. Lupus disease activity does not correlate with depression or other neuropsychiatric symptoms [6]. Another important neuropsychiatric manifestation prevalent in JSLE patients is cognitive impairment, which can affect 20% to 70% of patients, unrelated to disease activity or other manifestations [7,8,9]. Children and adolescents affected by SLE go through a different neuronal development process than their healthy counterparts, likely due not only to the inflammatory and vascular abnormalities secondary to autoimmune activation, but also to lupus treatments. Cognitive deficits that are frequently present in young JSLE patients may disturb attention, memory, and executive function, representing a challenge that may interfere with academic and social performance [9]. This study examined the influence of clinical factors and disease activity on depression and anxiety in adult JSLE patients, especially compared to adult-onset SLE. It also included control populations of patients with depression without autoimmune disease and healthy controls. In addition to comparing depressive patterns between these groups, individual factors were also considered for their effect on depression and anxiety in JSLE patients. These factors included psychological, social, and clinical values. We hypothesized that the patterns of depression and anxiety would be different between the juvenile- and adult-onset SLE patients, and that there would be underlying psychosocial or clinical factors that would help to explain the depression and anxiety in JSLE patients.

## 2. Materials and Methods

### 2.1. Population

This cohort study included 29 Portuguese Caucasian patients with JSLE, with at least 4 American College of Rheumatology Criteria (ACR) ([10]). For the purposes of this study, the JSLE group consists of individuals who were diagnosed as children, although their mean current age is 23. Control groups included 22 healthy subjects, 32 depressed control subjects, and 73 subjects with adult-onset systemic lupus erythematosus. All the patients were followed at the Rheumatology Outpatient Unit of São João Hospital. The JSLE patients were recruited between October 2018 and May 2019; laboratory evaluation was obtained at least one month prior to the psychosocial assessment. Written and verbal information about the study was presented to all eligible patients, and willingness to participate was the unique inclusion criteria applied.

The study was approved by the Ethical Committee of the São João Hospital IRB (EPE) in accordance with the Declaration of Helsinki, and informed written consent was obtained from each participant and/or legal guardian.

### 2.2. Psychosocial Evaluation

Psychosocial evaluation was conducted through the application of a battery of standardized instruments, as previously described [11,12,13]. Instruments include the Fatigue Severity Scale (FSS) [14,15,16,17], the Hospital Anxiety and Depression Scale (HADS) [18,19,20], and the Medical Outcomes Study Questionnaire Short Form 36 Health Survey (SF-36) [21,22].

### 2.3. Clinical and Laboratory Evaluation

Patient recruitment and access to clinical data were carried out during routine visits or consultations, as previously described [11,12,13]. A snowball approach was used to recruit the unaffected control population.

Laboratory and clinical evaluations were obtained for the JSLE and SLE patients through clinical records. Lab tests included leukocytes (10^9^/L), lymphocytes (percentage), platelets (10^9^/L), erythrocyte sedimentation rate (mm/h), anti-dsDNA antibody titer (IU/mL), C-reactive protein level (mg/dL), blood urea nitrogen (BUN) (mg/dL), creatinine (mg/dL), albumin (g/L), urine sediment, complement 3, 4, and anti-dsDNA, among others. Physical activity, smoking, and alcohol consumption were also recorded.

### 2.4. Statistical Analysis

Significant differences in the demographic, clinical, and psychological variables between the JSLE subjects, SLE subjects, healthy controls, and depressed subjects were determined using the independent *t*-test, Fisher’s exact chi-squared, Mann–Whitney U, Wilcoxon rank sum, or Welch’s tests when considered appropriate. Association of depression or anxiety to clinical, social, or other psychological factors was carried out using univariate and multivariate analysis, as previously described [11,12,13].

## 3. Results

### 3.1. Demographics

Patient’s sociodemographic and clinical characteristics are presented in (Table 1). As expected, the patients with JSLE were significantly younger than the patients with SLE, or any of the other groups (*p* < 0.001), although the criteria for inclusion in the JSLE group was pediatric-onset lupus, not childhood itself, and the mean age of these participants was 23. There were fewer female patients in the JSLE cohort than the SLE cohort, with the JSLE cohort consisting of 90% female participants and the SLE cohort being 100% female (*p* < 0.03). There was no significant difference between the female/male ratio in the JSLE group and the depressed subject group. The JSLE cohort was significantly more educated than any group other than the normal controls. For example, 87% of the JSLE group had some college or graduate study, while only 47% of the SLE group and 30% of the depressed patients had this level of education (*p* < 0.001 for each group).

### 3.2. Clinical Characteristics of the JSLE Patient Cohort

The lupus manifestations of the JSLE patient cohort, as well as the treatments they are receiving, is provided in Table 2. Notably, there is no significant difference in symptoms of depression or anxiety that correlate with the SLEDAI score, although there is a correlation with the SLICC score. This may be explained, since the SLEDAI measures active symptoms, while the SLICC measures more accumulated damage due to SLE. No other clinical manifestations were significantly associated with depression or anxiety.

Corticosteroid use and dose, as well as lisinopril use, were associated with symptoms of depression and anxiety. Hydroxychloroquine use inversely correlated with depressive symptoms but not anxiety, and pentoxifylline use was positively correlated with depression (Table 2).

### 3.3. Psychological Indicators between Groups

**Figure 1:**
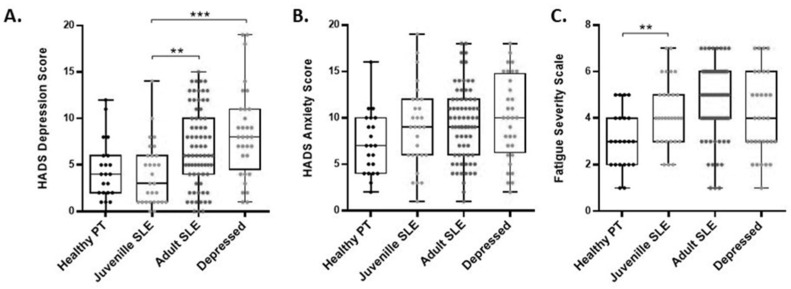
JSLE patients show fewer depressive symptoms than other patient groups, but similar anxiety and fatigue symptoms. Symptoms of depression and anxiety were measured using the HADS depression and anxiety scale. JSLE patients scored significantly lower on the depression symptoms than the SLE patients, or the depressed non-autoimmune patients. The mean JSLE depression score was 4.0, while the mean SLE score was 6.92 (**A**). Symptoms of anxiety were not significantly different between the JSLE patients and any other group (**B**). Fatigue in JSLE patients was significantly higher than in the healthy controls, but not significantly different from the SLE or depressed patient groups (**C**). Fatigue scores were significantly different between jSLE patients and healthy controls, but not significantly different between jSLE patients and other patient groups. ** *p* < 0.005, *** *p* < 0.0005 (independent *T*-test).

The HADS depression score showed that the JSLE patients exhibited lower depressive symptoms compared to every other group except for the healthy controls. They had a lower mean depression score (4.0) than the SLE group (6.92) and the depressed group (8.38) (*p* < 0.005 for each group). The JSLE patients did not have significantly different depression scores than the healthy controls (4.32, *p* = 0.735). With the cut-off for depression set at 8 on the HADS scale, only four of the 29 JSLE patients qualified as depressed when the survey was taken (Figure 1).

Anxiety, however, was high in the JSLE patients and did not significantly differ between the JSLE group and any of the other groups. The JSLE patients had a mean score of 9.03 on the HADS anxiety scale, while the SLE group had an anxiety score of 9.41, and the depressed patients had a score of 10.0. Using a score of 8 as a measure of moderate anxiety, 18/29 JSLE patients were positive for anxiety (Figure 1). All of the JSLE patients with depression also exhibited anxiety.

Fatigue consistently strongly correlated with depression and anxiety in the adult SLE patients we studied previously [11,13]. The JSLE patients, despite having lower depression scores than the SLE patients, had similar fatigue scores (4.28 in JSLE compared to 4.78 in SLE). JSLE patients had significantly higher fatigue than the healthy controls (3.09, *p* = 0.003). The fatigue scores of the JSLE patients were similar to those of the depressed patients (4.13) (Figure 1).

### 3.4. Clinical Comparisons between Groups

**Figure 2:**
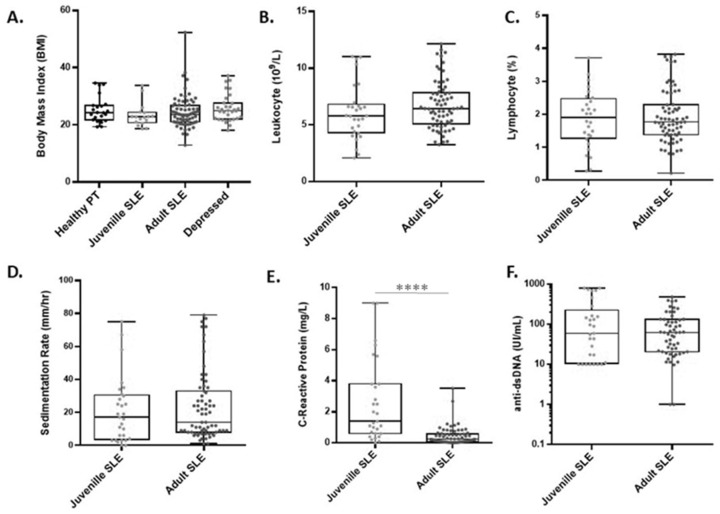
Clinical values are similar between JSLE and SLE patients. No statistical differences were found in nearly all clinical values between JSLE and SLE patients including (**A**) body mass index, (**B**) leukocyte counts, (**C**) lymphocyte count, (**D**) sedimentation rate, and (**F**) dsDNA levels. (**E**) C-reactive protein levels were significantly higher in JSLE patients compared to the adult SLE cohort. **** *p* < 0.0001 (Mann–Whitney U).

The values of the clinical tests between the JSLE group and the SLE group were very similar. There were no significant differences between these groups in leukocyte count, lymphocyte count, dsDNA antibody concentration, sedimentation rate, or any other clinical value measured in this study (Figure 2), save for C-reactive protein levels, which were significantly higher in the JSLE patients compared to the SLE subjects (Figure 2E) (*p* < 0.0001). As would be expected, the JSLE patients had significantly shorter disease duration (7.17 years) than the SLE patients (15.77 years, *p* < 0.001).

### 3.5. Factors Correlating with Depression and Anxiety in JSLE Patients

In order to understand depression and anxiety in the JSLE patients, survey instruments and clinical values were examined for correlation with anxiety and depression in the JSLE patients.

#### 3.5.1. Survey Results

Within the JSLE group, fatigue was strongly correlated with depressive symptoms (pseudo R^2^ = 0.07, *p* < 0.001) (Table 2). In our previous study of depression in SLE patients [11], we found pain to be strongly correlated with depressive symptoms, and this association remained in the JSLE group (pseudo R^2^ = 0.073, *p* < 0.001). As expected, subjective happiness was strongly inversely correlated with depression (pseudo R^2^ = 0.173, *p* < 0.001). In terms of the subjects’ perception of their health using the SF-36 instrument, depressive symptoms strongly correlated with poor general health (pseudo R^2^ = 0.043, *p* = 0.009). Depressive symptoms were also correlated with inability to perform physical roles (pseudo R^2^ = 0.030, *p* = 0.024) and emotional roles (pseudo R^2^ = 0.046, *p* < 0.001). Social function (pseudo R^2^ = 0.104, *p* < 0.001) and vitality (pseudo R^2^ = 0.091, *p* < 0.001) both inversely correlated with depressive symptoms (Table 3).

The psychological survey results that correlated with anxiety were in many ways similar to those for depression, with some notable exceptions. Fatigue was not significantly correlated with anxiety, nor was body pain. Although limitation in physical roles was not associated with anxiety, a limitation of emotional roles did show a correlation (pseudo R^2^ = 0.069, *p* < 0.001). Social function (pseudo R^2^ = 0.049, *p* = 0.003) and vitality (pseudo R^2^ = 0.073, *p* < 0.001) inversely correlated with anxiety.

#### 3.5.2. Correlations between Laboratory Values and Depression and Anxiety

**Figure 3:**
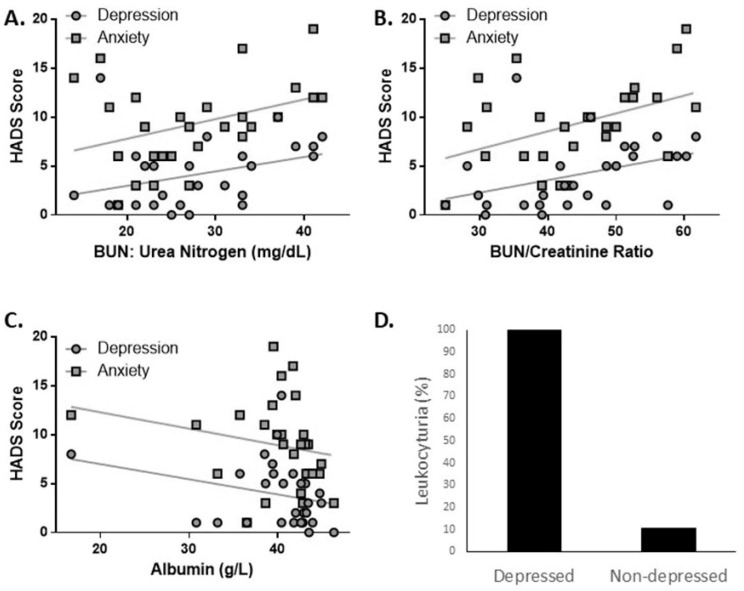
Kidney function shows multiple associations with symptoms of depression and anxiety in the JSLE patients. (**A**) BUN levels positively correlate with increased depression in JSLE patients (pseudo R^2^ = 0.056, *p* = 0.003 for depression, pseudo R^2^ = 0.066, *p* < 0.001 for anxiety). (**B**) Similarly, the BUN/Creatinine ratio in JSLE patients correlates with depression (pseudo R^2^ = 0.065, *p* = 0.002) and anxiety (pseudo R^2^ = 0.08, *p* < 0.001). (**C**) Albumin levels were inversely correlated with depression and anxiety in the JSLE patients (pseudo R^2^ = 0.035, *p* = 0.01). (**D**) Leukocyturia was found in all of the JSLE patients with depressive symptoms, but only 13% of non-depressed JSLE patients (χ^2^ = 15.75 *p* < 0.001). Statistical test for A, B, C was GLM logistic regression with the Poisson function. Significance of D was calculated using Fisher’s exact chi-square analysis.

The majority of the laboratory values that correlated with depression in the JSLE patients involved kidney function. Both the blood urea nitrogen (BUN) levels (pseudo R^2^ = 0.056, *p* = 0.003) and BUN/creatinine ratio (pseudo R^2^ = 0.065, *p* = 0.002) correlated with depressive symptoms. Both of these measures also correlated even more strongly with symptoms of anxiety (BUN pseudo R^2^ = 0.066, *p* < 0.001, BUN/creatinine ratio pseudo R^2^ = 0.080, *p* < 0.001). As a correlation between depression and/or anxiety could be confounded by corticosteroid use, since it is known that BUN/Cre can be increased by corticosteroid treatment, we compared the BUN/Cre levels between JSLE patients being treated with and without corticosteroids and found that their BUN/Cre ratios were not significantly different (*p* = 0.3830, independent *t*-test) with similar means at 42.89 and 37.48, respectively. The presence of leukocyturia was also associated with depressive symptoms. Every JSLE patient that showed depressive symptoms also had leukocyturia, in contrast with only 13% of the non-depressed patients (chi-square = 15.75, *p* = 6.99 × 10^−5^). Low albumin levels also significantly correlated with depressive symptoms (pseudo R^2^ = 0.035, *p* = 0.01). (Figure 3). Higher platelets correlated with increased depression (pseudo R^2^ = 0.058, *p* = 0.002) and anxiety (pseudo R^2^ = 0.032, *p* = 0.018).

There was no correlation between the majority of clinical tests, including common indicators such as the sedimentation rate or complement levels, and depression or anxiety (Table 4).

#### 3.5.3. Correlations between Treatments and Depression and Anxiety

The use of several medications was examined to determine if there was a correlation between medical treatment and symptoms of depression or anxiety. Both the presence of corticotherapy (OR = 1.08–2.96, *p* = 0.032) and the corticosteroid dosage (pseudo R^2^ = 0.072, *p* = 0.021) significantly correlated with depressive symptoms. The use of lisinopril also correlated with depressive symptoms (OR = 1.10–2.44, *p* = 0.013), as did the use of pentoxifylline (OR = 1.16–3.04, *p* = 0.007). Corticotherapy also correlated with anxiety (OR = 1.07–2.02, *p* = 0.02), as did the dose of corticosteroids used (pseudo R^2^ = 0.33, *p* = 0.019).

#### 3.5.4. Multivariate Analysis

Multivariate analysis was performed using the best fit model to predict the clinical laboratory factors that were most likely to predict depression and anxiety in JSLE. The BUN/creatinine ratio was the strongest predictor of both depression and anxiety in the multivariate analysis (*p* = 0.043 for depression and *p* < 0.001 for anxiety) (Table 5).

## 4. Discussion

Kidney function correlated with symptoms of depression and anxiety in the JSLE patients across several measures. The BUN/creatinine ratio was implicated in depression in both the univariate and the multivariate analysis. In fact, the BUN/creatinine ratio was indicated as the lab test most likely to predict symptoms of both depression and anxiety. Cells were present in the urine in every one of the depressed JSLE patients. Finally, low albumin was also associated with depression. Low serum albumin correlates with kidney disease [23] and outcomes in lupus nephritis [24]. These kidney findings are especially interesting given that none of the JSLE patients were diagnosed with lupus nephritis. The lack of diagnosis of lupus nephritis does not preclude kidney involvement in lupus, but it does mean that although some of the patients had kidney involvement, this did not rise to the level of diagnosed nephritis. These markers of kidney dysfunction may, therefore, be useful in predicting depression and anxiety even before they become severe enough for a diagnosis of nephritis.

The correlations between measurements involving the kidneys and depression or anxiety, taken together, suggest kidney markers could also serve as biomarkers for depression. The association could be present because the same processes that damage the kidneys also contribute to depression. For example, immune complex deposition could lead to systemic inflammation and inflammatory cytokine production, influencing depression and anxiety at the same time as kidney function. Similarly, autoantibodies that tend to affect the kidneys could also be involved in mediating depression. Another possibility is that kidney involvement signifies active, severe disease, which is more likely to be associated with depression. This is an interesting finding considering that the standard measures of disease activity, such as sedimentation rate and complement levels, were not significantly correlated with depression or anxiety, suggesting that organ-specific or symptomatic measures may be more valuable in predicting depression and anxiety in JSLE than traditional measurement of inflammation used to quantify disease activity.

The correlations between corticosteroid dose and depression and anxiety support the idea that more severe disease leads to more severe psychological symptoms. Patients with more severe disease are likely to be taking higher doses of medication. In this way, corticosteroid dose may act as another indicator of severity of disease that is not a traditional lab test. Corticosteroids themselves may also have an effect on psychiatric symptoms, and these can sometimes be difficult to untangle from neuropsychiatric symptoms of lupus [25].

Symptoms of depression were lower among JSLE patients overall than among the adult-onset SLE patients. Anxiety and fatigue, however, were not significantly different between the JSLE patients and the SLE patients. This is unusual because depression and anxiety tend to correlate very strongly, as do depression and fatigue. Indeed, fatigue was correlated with depression when the JSLE population was examined alone. The relative youth of the JSLE patients may be protective against depressive symptoms because of the shorter length of disease and possibly overall lower disease burden.

We can also hypothesize that educational achievement has a protective role for depression in the JSLE patients, since the JSLE group has significantly higher education than the adult SLE group. Higher education significantly decreases the odds for depression [26] with stronger effects in adolescence, a critical developmental period where relevant resources and self-efficacy are built [27,28]. Several pathways have been identified, linking higher education to lower levels of depression, namely the enhanced ability to cope with challenges and life events [29]. Lupus is a disabling disease affecting JSLE patients early in life, and the development of skills fostered by education may help patients to cope with the physical and psychological burden of the illness.

Prior correlations between depression and fatigue have left it unclear whether fatigue results in depression or depression results in fatigue. These findings, where fatigue is present even without depression, lend support to the idea that fatigue can be a biological consequence of lupus.

Although JSLE patients had lower depression scores than the SLE patients, it was still possible to analyze factors that contributed to depression and anxiety in these patients. Pain and fatigue strongly correlated with depression, a finding that has been consistent for every group we have examined. JSLE patients with low perceived social function and overall health were also more likely to have depressive symptoms.

Study limitations: Although we were able to find several correlations between depression, anxiety, and features of JSLE, the study would have benefitted from a larger sample size and more complete matching with the three control groups. A wider range of symptomatic patients, including those with lupus nephritis, would also have been helpful in exploring the role of kidney function in psychiatric symptoms of JSLE. Future work could focus on understanding the link between education and protection from depression in lupus, as well as increasing the sample size and diversity.

Our JSLE patients were screened for the presence of major neuropsychiatric manifestations. Although none were found, the presence of depressive and anxiety symptoms may be insufficient to determine the occurrence of major depressive episodes, a recognized early sign of neuropsychiatric involvement of the autoimmune disease. Additionally, the observational cross-sectional design diminished the causality relationship between variables. We are aware of the probable confounding role of medication, since it may have either a protective or deleterious influence in psychological manifestations.

Overall, this study found an intriguing correlation between kidney function and symptoms of depression and anxiety. Although the JSLE patients on average had lower scores for depressive symptoms than the SLE patients, the JSLE patients still suffered from anxiety similar to the adult SLE patients. Many of the components that contributed to depression and anxiety in the adult-onset SLE patients [11] also correlated with depression and anxiety in the JSLE patients, factors such as fatigue, pain, and social isolation.

## Figures and Tables

**Table 1:** Sociodemographic characteristics of the patient cohort.

*Characteristics*	Juvenile SLE Patients	Healthy Subjects		Adult SLE Patients		Depressed Patients	
	(*N* = 29)	(*N* = 22)	*p*-Value	(*N* = 73)	*p*-Value	(*N* = 32)	*p*-Value
Gender, no. (%)			0.625		0.021		1.000
Female	26 (90%)	21 (95%)		73 (100%)		29 (90%)	
Age, mean ± SD	23 ± 5.4	43 ± 11.5	<0.001	45 ± 10.4	<0.001	49 ± 14.0	<0.001
Education Level, no. (%)			0.593		<0.001		<0.001
Primary	0 (0%)	0 (0%)		17 (23%)		8 (25%)	
Middle School	1 (3%)	1 (5%)		11 (15%)		7 (21%)	
High School	2 (7%)	2 (9%)		11 (15%)		7 (21%)	
College	16 (55%)	8 (36%)		24 (33%)		5 (15%)	
Graduate	10 (34%)	11 (50%)		10 (14%)		5 (15%)	

Statistical tests used: Welch’s two sample *T*-test, Fischer’s exact chi-squared test.

**Table 2:** Clinical manifestations and treatment of the JSLE patient cohort.

*Characteristics*		HADS Depression					HADS Anxiety				
	(*n* = 29)	Pseudo R^2^	AICc	Coefficient	Odds Ratio (95% CI)	*p*-Value	Pseudo R^2^	AICc	Coefficient	Odds Ratio (95% CI)	*p*-Value
***Clinical Exam Results***											
Age at Diagnosis, median (range)	16 (12–20)	<0.001	167.26	−0.016	0.98 (0.90–1.07)	0.701	0.003	181.31	−0.019	0.98 (0.93–1.04)	0.500
Disease Duration (y), median (range)	5 (1–23)	0.011	165.57	0.023	1.02 (0.99–1.06)	0.167	0.013	179.41	0.017	1.02 (1.00–1.04)	0.119
Body Mass Index	23.43 ± 3.75	0.005	166.62	0.021	1.02 (0.97–1.07)	0.364	0.015	179.15	0.026	1.03 (1.00–1.06)	0.097
SLEDAI, median (range)	2 (0–12)	<0.001	167.41	0.001	1.00 (0.95–1.05)	0.978	0.003	181.16	0.014	1.01 (0.98–1.05)	0.432
SLICC, median (range)	0 (0–2)	0.043	160.39	0.435	1.54 (1.13–2.06)	0.004	0.019	178.46	0.215	1.24 (0.98–1.54)	0.059
Articular Manifestations, no. (%)	15 (52%)	0.017	164.59	0.315	1.37 (0.95–2.00)	0.096	0.006	180.66	−0.130	0.88 (0.69–1.12)	0.293
Mucocutaneous Manifestations, no. (%)	24 (83%)	0.015	165.03	0.417	1.52 (0.90–2.77)	0.143	0.024	177.47	0.369	1.45 (1.02–2.12)	0.048
Hematological Manifestations, no. (%)	13 (45%)	0.008	166.14	−0.212	0.81 (0.55–1.17)	0.264	0.002	181.46	−0.069	0.93 (0.73–1.19)	0.581
Renal Manifestations, no. (%)	12 (41%)	0.010	165.83	0.056	1.06 (0.97–1.15)	0.206	0.026	177.24	0.063	1.07 (1.00–1.13)	0.032
***Disease Management***											
Corticotherapy	22 (76%)	0.032	162.22	0.550	1.73 (1.08–2.96)	0.032	0.033	175.89	0.375	1.46 (1.07–2.02)	0.020
Dosage (mg), mean ± SD	7.78 ± 8.97	0.072	155.75	0.021	1.02 (1.00–1.04)	0.021	0.102	163.70	0.015	1.01 (1.00–1.03)	0.019
Mycophenolic Acid	8 (28%)	0.002	167.03	0.126	1.13 (0.75–1.67)	0.533	0.020	178.29	0.249	1.28 (0.99–1.65)	0.059
Lisinopril	6 (21%)	0.035	161.63	0.505	1.67 (1.10–2.44)	0.013	0.043	174.14	0.392	1.48 (1.12–1.93)	0.004
Hydroxychloroquine	26 (90%)	0.066	156.70	−0.816	0.44 (0.29–0.71)	<0.001	0.010	179.94	−0.257	0.77 (0.55–1.13)	0.163
Pentoxifylline	3 (10%)	0.039	161.10	0.650	1.92 (1.16–3.01)	0.007	0.003	181.16	0.151	1.16 (0.78–1.66)	0.430
Azathioprine	5 (17%)	0.017	164.68	0.376	1.46 (0.93–2.21)	0.087	0.005	180.89	0.149	1.16 (0.85–1.56)	0.341

**Table 3:** Univariate correlation of juvenile SLE patient psychosocial examination scores with the hospital anxiety and depression scale scoring.

*Characteristics*			HADS Depression					HADS Anxiety				
	(*n* = 29)	Reference (Score Range)	Pseudo R^2^	AICc	Coefficient	Odds Ratio (95% CI)	*p*-Value	Pseudo R^2^	AICc	Coefficient	Odds Ratio (95% CI)	*p*-Value
Fatigue Severity Scale, mean ± SD	4.28 ± 1.38	0 (0–7)	0.070	156.05	0.232	1.26 (1.10–1.44)	<0.001	0.019	178.40	0.083	1.09 (0.99–1.19)	0.067
Subject Happiness Scale, mean ± SD	5.20 ± 1.04	>20 (4–28)	0.173	139.22	−0.445	0.64 (0.55–0.75)	<0.001	0.0512	172.67	−0.176	0.84 (0.75–0.94)	0.002
General Health	48.62 ± 19.73	(0–100)	0.043	160.39	−0.013	0.99 (0.98–1.00)	0.009	0.048	173.22	−0.009	0.99 (0.98–1.00)	0.004
Reported Health Transition	58.62 ± 28.56	(0–100)	0.012	165.45	−0.005	1.00 (0.99–1.00)	0.160	0.005	180.96	−0.002	1.00 (0.99–1.00)	0.368
Physical Function	86.38 ± 15.46	(0–100)	0.017	164.60	−0.009	0.99 (0.98–1.00)	0.082	0.008	180.32	−0.005	1.00 (0.99–1.00)	0.221
Physical Role Limitation	81.48 ± 21.07	(0–100)	0.030	162.54	−0.009	0.99 (0.98–1.00)	0.024	0.003	181.32	−0.002	1.00 (0.99–1.00)	0.503
Emotional Role Limitation	77.87 ± 24.63	(0–100)	0.046	159.88	−0.010	0.99 (0.98–1.00)	0.005	0.069	169.62	−0.009	0.99 (0.99–1.00)	<0.001
Social Function	75.86 ± 22.14	(0–100)	0.104	150.46	−0.016	0.98 (0.98–0.99)	<0.001	0.049	172.99	−0.008	0.99 (0.99–1.00)	0.003
Vitality	53.68 ± 18.93	(0–100)	0.091	152.62	−0.019	0.98 (0.97–0.99)	<0.001	0.073	168.87	0.003	0.99 (0.98–0.99)	0.000
Mental Health	70.16 ± 17.08	(0–100)	0.178	138.44	−0.031	0.97 (0.96–0.98)	<0.001	0.208	144.87	-0.023	0.98 (0.97–0.98)	<0.001
Bodily Pain	53.10 ± 7.61	(0–100)	0.073	155.57	0.045	1.05 (1.02–1.07)	<0.001	<0.001	181.61	0.003	1.00 (0.99–1.02)	0.698

Statistical test used: GLM logistic regression with the Poisson function.

**Table 4:** Univariate correlation between clinical values and symptoms of depression and anxiety in JSLE subjects.

*Characteristics*		HADS Depression					HADS Anxiety				
	(*n* = 29)	Pseudo R^2^	AICc	Coefficient	Odds Ratio (95% CI)	*p*-Value	Pseudo R^2^	AICc	Coefficient	Odds Ratio (95% CI)	*p*-Value
***Clinical Laboratory Results***											
Hemoglobin (g/dL)	13.21 ± 1.40	<0.001	167.35	−0.016	0.98 (0.86–1.12)	0.814	0.007	180.60	0.007	1.05 (0.96–1.14)	0.279
Leukocytes (10^9^/L)	6.12 ± 2.46	0.010	165.82	0.048	1.05 (0.97–1.13)	0.203	0.013	179.44	0.038	1.04 (0.99–1.09)	0.124
Lymphocytes (10^9^/L)	1.85 ± 0.86	0.033	162.12	−0.002	1.00 (0.80–1.24)	0.984	0.024	177.53	−0.032	0.97 (0.84–1.12)	0.669
Platelets (10^9^/L)	247.90 ± 69.39	0.058	158.02	0.004	1.00 (1.00–1.01)	0.002	0.031563	176.17	0.032	1.00 (1.00–1.00)	0.018
Sedimentation Rate, ESR (mm/h)	20.86 ± 19.76	<0.001	167.31	0.002	1.00 (0.99–1.01)	0.749	<0.001	181.76	0.000	1.00 (0.99–1.01)	0.980
C-Reactive Protein, CRP (mg/L)	2.59 ± 2.63	<0.001	167.36	−0.008	0.99 (0.92–1.06)	0.818	0.004	181.11	−0.020	0.98 (0.93–1.03)	0.425
Albumin (g/L)	38.61 ± 9.33	0.035	161.78	−0.020	0.98 (0.97–1.00)	0.010	0.017	178.80	−0.011	0.99 (0.98–1.00)	0.071
Anti-dsDNA (IU/mL)	197.54 ± 272.99	<0.001	167.25	0.000	1.00 (1.00–1.00)	0.685	<0.001	181.76	0.000	1.00 (1.00–1.00)	0.937
C3 (mg/dL)	99.95 ± 22.33	<0.001	167.39	−0.001	1.00 (1.00–1.01)	0.889	0.001	181.51	0.001	1.00 (1.00–1.01)	0.612
C4 (mg/dL)	16.21 ± 7.72	0.012	165.50	−0.018	0.98 (0.96–1.01)	0.164	<0.001	181.69	−0.002	1.00 (0.98–1.01)	0.787
BUN: Urea Nitrogen (mg/dL)	25.76 ± 10.48	0.056	158.20	0.029	1.03 (1.01–1.05)	0.003	0.066	170.13	0.021	1.02 (1.01–1.03)	<0.001
Creatinine (mg/dL)	0.63 ± 0.13	<0.001	167.40	−0.083	0.92 (0.22–4.02)	0.911	<0.001	181.64	−0.175	0.84 (0.32–2.22)	0.723
BUN/Creatinine Ratio	41.21 ± 15.15	0.065	156.83	0.023	1.03 (1.01–1.04)	0.002	0.080	167.57	0.017	1.02 (1.01–1.03)	<0.001
Urine (abnorml), no. (%)	14 (48%)	0.008	166.17	0.207	1.23 (0.85–1.78)	0.266	0.005	180.90	0.115	1.12 (0.88–1.43)	0.353
Proteinuria, no. (%)	13 (93%)										
Proteinuria (g/dL), mean ± SD	0.72 ± 0.94	0.471	91.48	−0.059	0.94 (0.67–1.26)	0.713	0.522	89.98	0.037	1.04 (0.85–1.24)	0.700
Leukocyturia, no. (%)	7 (50%)										
Leukocyturia (cells/uL), mean ± SD	326.14 ± 463.21	0.743	48.91	−0.002	1.00 (0.99–1.00)	0.033	0.823	38.32	−0.001	1.00 (1.00–1.00)	0.033
Erythrocyturia, no. (%)	2 (14%)										
Erythrocyturia (cells/uL), mean ± SD	282.15 ± 310.77	0.962	−1.84	0.005	1.01 (1.00–1.01)	0.028	0.958	<0.001	0.002	1.00 (1.00–1.01)	0.121

Statistical test used: GLM logistic regression with the Poisson function.

**Table 5:** Multivariate model of clinical predictors of depression and anxiety for the juvenile SLE subjects.

Depression			
Variable	Coefficient	Odds Ratio (95% CI)	*p*-value
BUN/Creatinine Ratio	0.021	1.02 (1.00–1.04)	0.043
**Model Fit Summary**		Pseudo R^2^	AICc
		0.513	90.21
**Anxiety**			
Variable	Coefficient	Odds Ratio (95% CI)	*p*-value
BUN/Creatinine Ratio	0.018	1.02 (1.01–1.03)	<0.001
**Model Fit Summary**		Pseudo R^2^	AICc
		0.137	162.71

## Data Availability

Data collected for this study is available upon request to the corresponding author.
